# Drugs of Dependency: The Pregnant Woman and Her Infant

**DOI:** 10.1155/2011/719894

**Published:** 2012-04-11

**Authors:** Julee Oei, Anne Bartu, Lucy Burns, Mohamed E. Abdel-Latif, Chulathida Chomchai

**Affiliations:** ^1^Department of Newborn Care, Royal Hospital for Women, Barker Street, Randwick, NSW 2031, Australia; ^2^School of Nursing and Midwifery, Faculty of Health Sciences, Curtin University, Perth, WA 6102, Australia; ^3^National Drug and Alcohol Research Centre, The University of New South Wales, Sydney, NSW 2052, Australia; ^4^Department of Neonatology, The Canberra Hospital, Yamba Drive, Garran, ACT 2605, Australia; ^5^Science Division, Mahidol University International College, Salaya 73170, Thailand

The repercussions of drug abuse are particularly emphasized when a pregnant woman is affected. Gestational drugexposure is associated with significantly increased risks of poor maternal health, adverse perinatal outcomes, and unfavourable psychosocial consequences. Children affected by maternal drug use are at particular risk form their parent's drug seeking behavior as well as the toxicological effects of the drugs used. Early identification is key, and considerably more research is needed to develop the optimum means by which affected mother-infant dyads may be recognized and supported. In this special issue, we present six papers that deal with the problems caused by gestational drugs of dependency. 

“*Pregnant and non-pregnant women in cape town, South Africa: drug use, sexual behavior, and the need for comprehensive services*” deals with the problem of methamphetamine use in pregnant women in South Africa, a country where poverty and HIV risk are high. The authors find that methamphetamine, a drug highly associated with adverse psychosocial sequelae, is proportionally used by more pregnant than non-pregnant women, a practice that could have far-reaching effects on their families. 

“*Drug testing for newborn exposure to illicit substances in pregnancy: pitfalls and pearls*” in this special issue provides an overview of drug testing of newborns. Accurate recognition of a newborn whose mother has used illicit drugs during pregnancy influences decisions regarding healthcare of mother and infant whilst in hospital and following discharge. The difference between screening and confirmatory drug testing and the potential for false-positive results by immunoassay screening are discussed. Testing of newborns for illicit drugs can be done on urine, blood, meconium, hair, and umbilical cord blood or tissue samples. The implications and limitations of drug testing are presented. The authors caution that illicit drug use in pregnancy is not an independent predictor of a mother's ability to adequately care for her child but needs to be considered in health care planning.

“*Initial feasibility of a woman-focused intervention for pregnant African-American women*” reports on the difficulties faced by drug-using women in accessing antenatal care and contraception compared to the general pregnant population. The authors discuss challenges faced in the development of innovative and effective systems to educate affected women and to provide adequate care for the women and their children. 

“*Pharmacological treatment of neonatal opiate withdrawal: between the devil and the deep blue sea*” deals with the issues faced by clinicians on how to optimize treatment of the newborn infant withdrawing from maternal opiates. They emphasize that current clinical research and evidence on the long term development of children affected by in utero opiate exposure is limited as are the effects of pharmacological treatment on these infants, especially when there is an increasing body of evidence suggesting that opiates may not have a benign influence on fetal or, indeed, neonatal neurodevelopment. 

“*Psychosocial characteristics and obstetric health of women attending a specialist substance use antenatal clinic in a large metropolitan hospital*” investigates the outcomes of a community-based intervention to improve drug-use behavior and social circumstances of crack-using African-American women. It highlights, in particular, the benefits of training in social skills such as relationship support to alleviate lifestyle issues associated with highly dependent drug-use behaviors. 

“*Effectiveness of a smoking cessation intervention for methadone-maintained women: a comparison of pregnant and parenting women*” examines the effects of an intervention aimed to reduce cigarette smoking for women in substance abuse programs. This is particularly important as cigarette smoking compounds the risk of adverse perinatal and neonatal outcomes in this population, and the paper highlights the benefits of addressing what would often be considered a trivial problem for the drug-using woman. 

*Julee Oei* *Julee Oei* *Anne Bartu* *Anne Bartu* *Lucy Burns* *Lucy Burns* *Mohamed E. Abdel-Latif* *Mohamed E. Abdel-Latif* *Chulathida Chomchai* *Chulathida Chomchai*

